# Supplementary material for: Solid-Phase Synthesis of Red Fluorescent Carbon Dots for the Dual-Mode Detection of Hexavalent Chromium and Cell Imaging
Source: Biosensors (Basel). 2022 Jun 20;12(6):432. doi: 10.3390/bios12060432 (PMC9221384; doi:10.3390/bios12060432)
Supplement: Supplementary file 1 [file biosensors-12-00432-s001.zip › biosensors-1749120-supplementary.pdf]

Article

Solid-phase Synthesis of Red Fluorescent Carbon Dots for the Dual-mode Detection of Hexavalent Chromium and Cell Imaging

Jinshuang Hu [1], Xin Wang [1], Hua Wei [1], Lei Zhao [2,*], Boxuan Yao [1], Caiyun Zhang [1], Jiarui Zhou [1], Jian Liu [3], Shenghong Yang [1,*]

[1] Shandong Provincial Key Laboratory of Molecular Engineering, School of Chemistry and Chemical Engineering, Qilu University of Technology (Shandong Academy of Sciences), Jinan, 250353, P.R. China.

[2] Key Laboratory of Biotechnology and Bioengineering of State Ethnic Affairs Commission, Biomedical Research Center, Northwest Minzu University, Lanzhou, 730030, China.

[3] Institute of Advanced Materials, Jiangxi Normal University, Nanchang, 330022, China.

* yangsh@qlu.edu.cn (S.H. Yang), zhaolei@xbmu.edu.cn (L. Zhao)

Figures

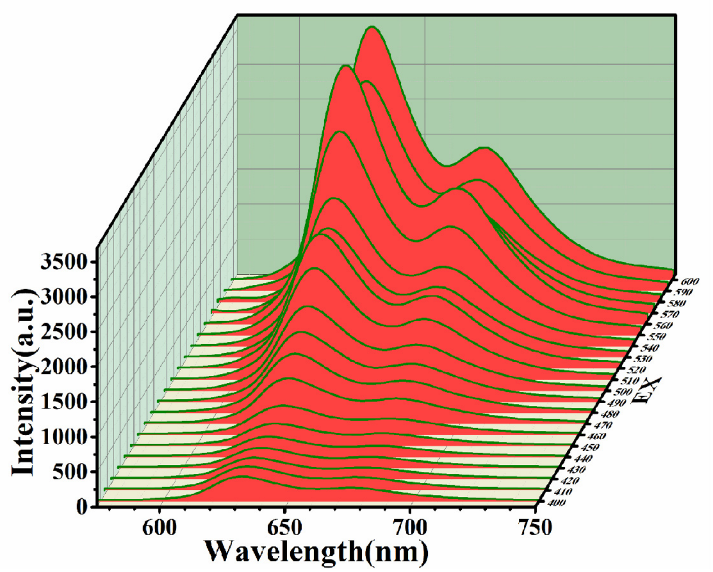

Figure S1. The PL spectra of R-CDs with excitation wavelength from 400 to 600 nm.

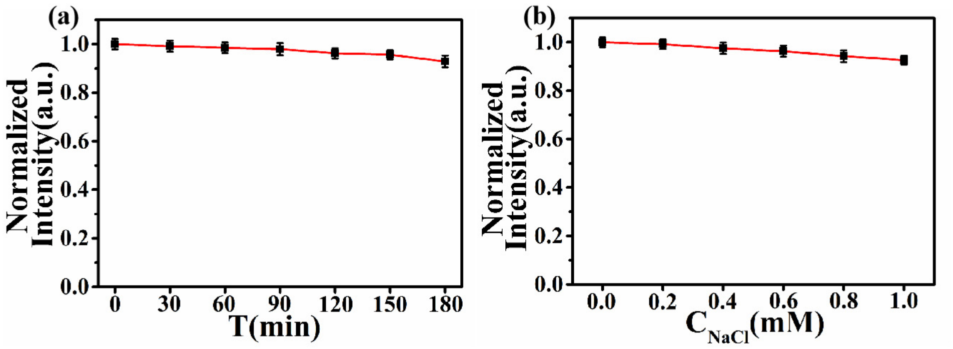

Figure S2. Influence of light illumination (a) and ion strength (b) on the PL intensity of R-CDs.

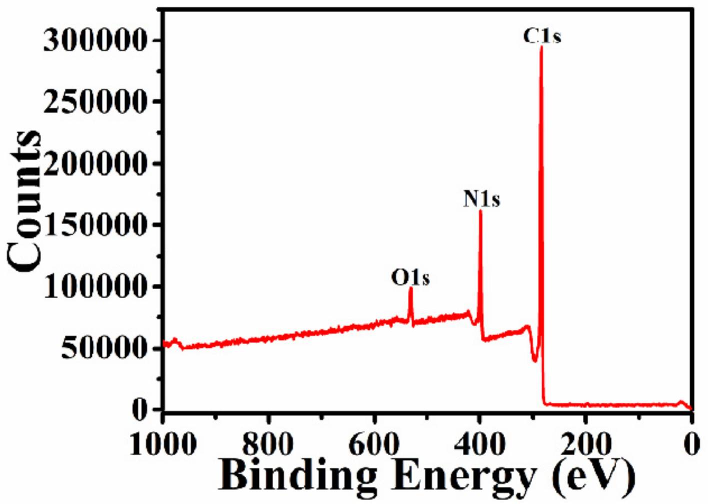

Figure S3. XPS survey spectra of synthesized R-CDs.

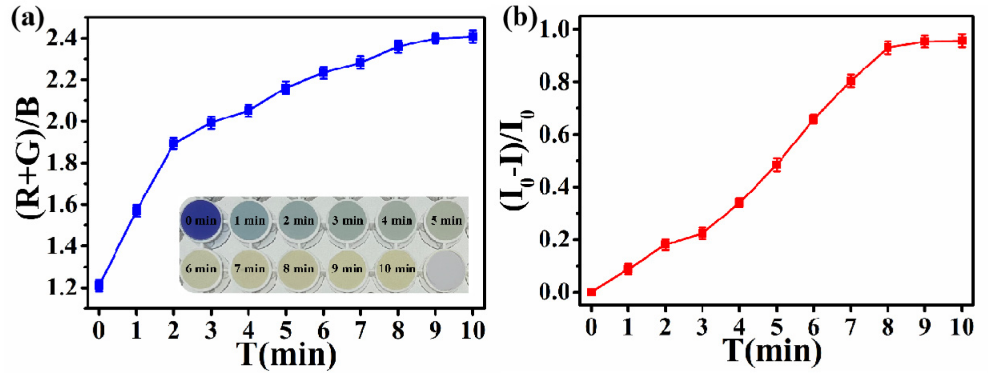

Figure S4. The R ,G ,B values (a) and fluorescent signal (b) response time of R-CDs to Cr(VI), Inset: pictures of 96-well plates with different response times of Cr(VI).

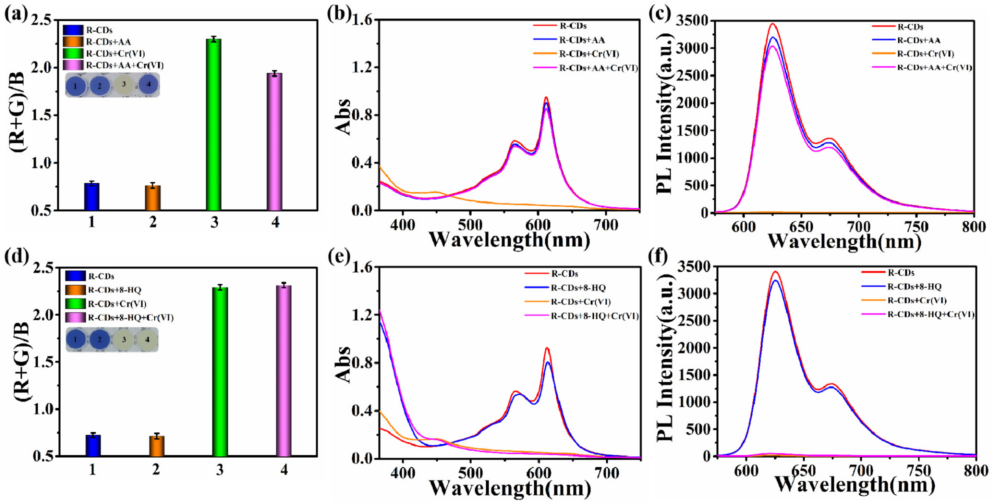

Figure S5. The influence of ascorbic acid (AA) on the scanometric, absorption and fluorescent sensing of Cr(VI), the influence of 8-hydroxyquinoline (8-HQ) on the scanometric, absorption and fluorescent sensing of Cr(VI).

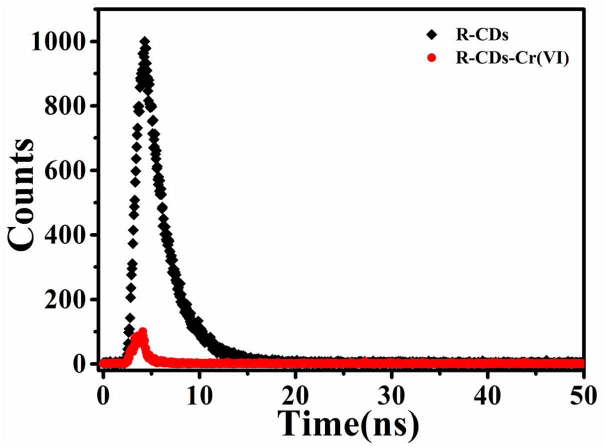

Figure S6. Fluorescence lifetime of R-CDs and R-CDs-Cr(VI).

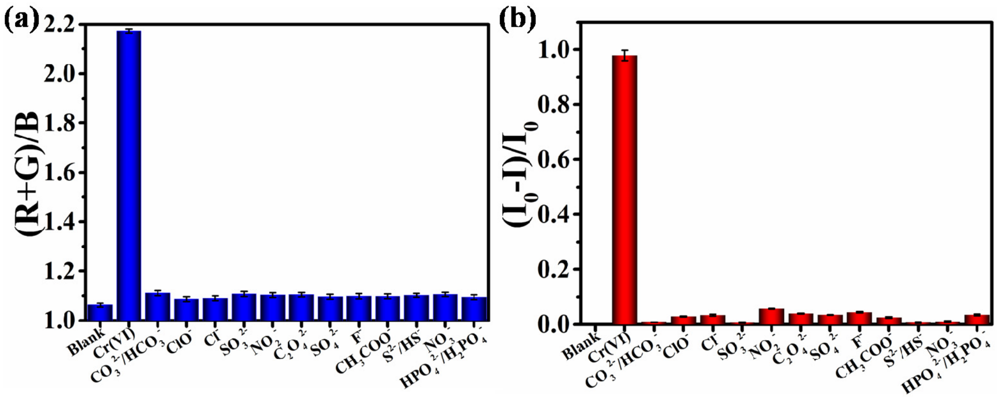

Figure S7. The change of (R+G)/B (a) values of R-CDs for various anions and the response of luminescence intensity $(I_0\text{-}I)/I_0$ (b). The colorimetric and fluorescence mode concentrations of all heavy metal anions are 50 μM.

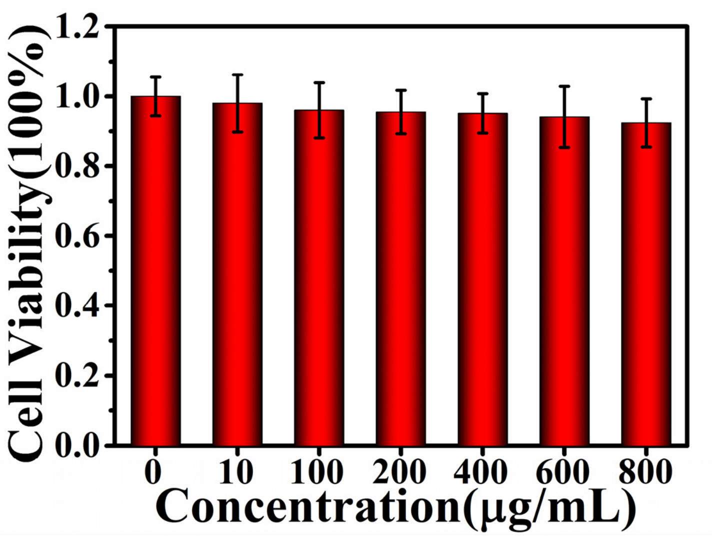

Figure S8. MTT test results of R-CDs.

Table S1. Values (%) of each peak fitted.

Element	Peak Fitted	Value (%)
C	C=O	34.2
	C-O/C-N	33.5
	C-C/C=C	32.3
N	Pyrrole N	5.3
	Amine N	53.4
	Pyridine N	41.3
O	C=O	34.2
	C-O	65.8

Table S2. Comparison of different probes for detection of Cr(VI).

Sensor	Fluorometry (μM)	Colorimetry (μM)	Ref.
N-CQDs	2.1	-	1
NH_2-CuMOFs	0.018	-	2
N, B-CQDs	0.077	-	3
CP-CDs	0.015	-	4
CDs	0.19	-	5
PEG	0.75	-	6
CNDs	0.058	-	7
NH_2-mSiO_2@CDs	0.005	-	8
CDs@Eu-MOFs	0.21	-	9
USTS-7	0.21	-	10
BNCDs	0.41	-	11
CMC-SQDs	0.024	-	12
N-CDs	0.023	-	13
N-CDs-W-THF	-	0.3	14
Au NDC@Ag NRs	-	1.69	15
$CoFe_2O_4$/H_2PPOP	-	0.026	16
CDs	0.26	0.062	17
GQDs	0.43	0.10	18
B/Y-CQDs	0.04	0.55	19
CDs	0.14	0.041	20
**R-CDs**	**0.009**	**0.098**	**This work**

Table S3. The fluorescence method is used to detect Cr(VI) in actual water samples.

Sample	Spiked (μM)	Found (μM)	Recovery (%)	RSD (%)
Tap water	0.1	0.097	97.3	1.2
	0.5	0.48	96.6	4.1
	1.5	1.53	102.3	1.6
Rain water	0.1	0.106	106.0	1.2
	0.5	0.49	98.6	2.5
	1.5	1.47	97.9	3.4
Pearl Spring	0.1	0.103	103.5	1.8
	0.5	0.53	106.3	2.9
	1.5	1.39	92.6	3.1
Daming Lake	0.1	0.098	98.4	4.3
	0.5	0.52	104.6	2.4
	1.5	1.532	102.1	1.7
